# Identification of risk factors for post-endoscopic retrograde cholangiopancreatography pancreatitis in a high volume center

**DOI:** 10.1371/journal.pone.0177874

**Published:** 2017-05-17

**Authors:** Veit Phillip, Miriam Schwab, David Haf, Hana Algül

**Affiliations:** Klinik und Poliklinik für Innere Medizin II, Klinikum rechts der Isar, Technische Universität München, München, Germany; University of Szeged, HUNGARY

## Abstract

**Background/Objectives:**

Pancreatitis is the most common complication of endoscopic retrograde cholangiopancreatography (ERCP). Several patients´ or procedure related risk factors for post-ERCP pancreatitis (PEP) have been suggested. The aim of this study was to validate the risk factors for PEP in a high-volume center.

**Methods:**

All patients undergoing first time ERCP at a tertiary referral center between December 2010 and October 2013 were retrospectively included. PEP was defined according to the Atlanta Classification.

**Results:**

404 patients were included in the final analysis. The risk to develop PEP was increased in patients after inadvertent cannulation of the pancreatic duct (odds ratio 7.468 (2.792–19.975); p<0.001), which occurred in 37.4% of the patients. Inadvertent cannulation occurred significantly more frequently in patients with difficult cannulation of the papilla duodeni major (odds ratio 7.3; p<0.001).

**Conclusion:**

Inadvertent cannulation of the pancreatic duct is a procedure related risk factor for PEP. Measurements on preventing inadvertent cannulation of the pancreatic duct should be established and studies on prophylactic measurements should focus particularly on patients with inadvertent cannulation of the pancreatic duct.

## Introduction

Endoscopic retrograde cholangiopancreatography (ERCP) is a well-established therapeutic procedure for benign and malignant diseases of the biliopancreatic system. However, post-ERCP pancreatitis (PEP) is the most frequent complication of this procedure with an incidence of about 3–4% in unselected patients and up to 15% in high-risk patients [1–3]. Although most of the cases take a mild or moderate course, about 10% of all patients with PEP develop severe pancreatitis with an estimated pancreatitis-related mortality rate of 3% [1].

Over the last decades, several risk factors for PEP have been suggested. Female sex, young age, non dilated common bile duct, sphincter oddi dysfunction and previous incidence of pancreatitis are considered to be patient related risk factors [4–8]. Furthermore, difficult cannulation of the papilla and procedures associated with cannulation of the pancreatic duct such as pre-cut papillotomy and injection of contrast agent into the pancreatic duct seem to be procedure related risk factors [5–7, 9, 10].

Generally, mechanical trauma of the papilla during ERCP can lead to an edema or spasm of the sphincter of oddi and subsequently restrain the outlet of pancreatic juice thereby causing an elevation of the intrapancreatic pressure. As a consequence, pancreatic secretion is forced to the surrounding pancreas parenchyma, causing autodigestion, a critical event in the pathophysiology of acute pancreatitis [11, 12]. Moreover, cannulation of the pancreatic duct can lead to a damage of the epithelium [13]. In addition to the mechanical and hydrostatic damage, chemical, enzymatic, microbiological, allergic, and thermal factors can further favor PEP [14, 15].

The aim of this study was to validate the supposed and to possibly identify new risk factors for PEP in a high volume tertiary referral center.

## Patients and methods

All patients undergoing first time ERCP at the department of endoscopy between December 2010 and October 2013 were included in the study. The endoscopic database, medical charts and laboratory data were analyzed retrospectively.

First time ERCP was performed in 463 patients. 37 patients were excluded due to intended cannulation of the pancreatic duct for pancreatolithiasis, stenosis of the pancreatic duct, or suspected IPMN (intraductal papillary mucinous neoplasm). Further, 22 patients who suffered from acute pancreatitis at the time of ERCP were also excluded. This resulted in 404 patients which were included into the final analysis. The study was conducted in accordance with the Declaration of Helsinki and was approved by the local ethics committee (Ethikkommission der Medizinischen Fakultät der Technischen Universität München, project number 321/14). The ethics committee waived the need for written informed consent for this retrospective study. The number of ERCPs during the 35-month study period was 1425 in total which were performed by twelve endoscopists. Therefore, the average number of yearly ERCP per endoscopist was about 40.

Statistical analyses were performed using IBM SPSS Statistics 23 (SPSS Inc., Chicago, Illinois, USA). For descriptive data, mean ± standard deviation was used for normally distributed data and median (range; interquartile range, IQR) for not normally distributed data. For explorative data analysis, a chi-square test was used to calculate the odds ratio or spearman’s correlation to evaluate a statistical relation of suspected risk factors and PEP, inadvertent cannulation of the pancreatic duct, and difficult cannulation of the papilla duodeni major, respectively. To calculate correlations between continuous risk factors and dichotomous outcome we used a point biserial correlation. P-values < 0.05 were considered to be statistically significant.

Levels of difficulty for cannulation of the papilla duodeni major were categorized as easy (1–5 attempts) and difficult (>5 attempts) cannulation. PEP was defined according to the Atlanta Classification [16].

## Results

In total, 404 patients were included in the final analysis. Patients´ characteristics are shown in Table 1.

**Table 1:** Patients´ characteristics.

Sex, female (n = 404)	191/404 (47.3%)
Age (n = 404)	67.0 (17.0–94.0; 56.0–75.0)
Body weight, kg (n = 309)	74.0 (36.5–185.0;62.0–84.0)
Body height, cm (n = 305)	171±10
BMI, kg/m2 (n = 302)	24.9 (15.6–64.0;21.8–28.4)
**Indication for ERC** (n = 404)	
Bile duct stones	166 (41.1%)
Jaundice of unknown origin	112 (27.7%)
malignant bile duct stricture	74 (18.3%)
unknown bile duct stricture	19 (4.7%)
benign bile duct stricture	12 (3.0%)
others	21 (5.2%)

Data are presented as number (%), mean ± standard deviation, or median (range; IQR) as applicable. ERC, endoscopic retrograde cholangiography.

### Incidence of PEP

PEP occurred in 9.7% (39/404) of all patients. One case was classified as severe pancreatitis, all others as mild pancreatitis.

Incidence of PEP in patients with intended cannulation of the pancreatic duct was 16.2% (6/37).

### Risk factors for PEP

In univariate analyses, only inadvertent cannulation of the pancreatic duct was statistically significantly associated with PEP (odds ratio 4.373 (2.142–8.927); p<0.001). All data are presented in Table 2.

**Table 2:** Risk factors for post-ERCP pancreatitis.

Risk factor	p-value	odds ratio (CI 95%)
Female sex	0.627	0.848 (0.436–1.650)
Age	0.905	Correlation coefficient -0.006
Age < 40	0.571	1.438 (0.407–5.073)
Body weight, Kg	0.067	Correlation coefficient -0.104
Body height, cm	0.530	Correlation coefficient -0.036
BMI kg/m^2^	0.062	Correlation coefficient -0.107
BMI ≥ 25 kg/m^2^	0.261	0.638 (0.291–1.402)
Previous pancreatitis	0.384	1.744 (0.210–14.459)
Chronic pancreatitis	0.404	1.919 (0.405–9.091)
Status post cholecystectomy	0.676	1.191 (0.523–2.710)
Juxtapapillary diverticulum	0.640	0.773 (0.263–2.274)
Serum bilirubin <1.3 mg/dL	0.806	1.095 (0.530–2.264)
Bile duct diameter < 8 mm	0.678	0.834 (0.353–1.969)
ERCP on call	0.690	0.660 (0.084–5.157)
Difficult intubation of papilla	0.525	1.239 (0.639–2.403)
Inadvertent cannulation of the pancreatic duct	<0.001*	4.373 (2.142–8.927)
Transpancreatic papillotomy	0.149	1.753 (0.811–3.787)
Needle-knife papillotomy	0.746	0.713 (0.091–5.598)
Incomplete bile stone removal	0.873	1.108 (0.318–3.860)
Diagnosis bile duct stones	0.163	1.598 (0.824–3.102)
Diagnosis bile duct stricture	0.456	0.767 (0.382–1.542)
Prophylactic use of Diclofenac	0.949	0.934 (0.116–7.498)
Pancreatic duct stent	0.168	1.790 (0.775–4.131)

For dichotomous data, a chi-square test was performed. Point biserial correlations were used continuous data. P-values < 0.05 are considered statistically significant and are indicated by *. CI, confidence interval; BMI, body mass index; ERCP, endoscopic retrograde cholangiopancreatography.

Multivariate binary logistic regression analysis including the factors age, chronic pancreatitis in the medical history, normal serum bilirubin, non-dilated extrahepatic bile duct, cannulation of the pancreatic duct, and difficult cannulation of the papilla confirmed inadvertent cannulation of the pancreatic duct (odds ratio 7.468 (2.792–19.975); p<0.001) as an independent risk factor for PEP [17, 18]. All 12 patients diagnosed with chronic pancreatitis had an early stage of the disease. The median time period from first diagnosis of chronic pancreatitis to first time ERCP was 6.0 month (IQR, 0–60 month; range 0–102 month). 9 out of 12 patients (75%) had no exocrine or endocrine insufficiency.

### Risk factors for inadvertent cannulation of the pancreatic duct

Inadvertent cannulation of the pancreatic duct occurred in 151/404 (37.4%) of the patients. The risk to develop PEP was statistically significantly increased after inadvertent cannulation of the pancreatic duct (odds ratio 4.373; 95% CI 2.142–8.927; p<0.001)

Difficult cannulation of the papilla duodeni major, which means more than 5 attempts to intubate the papilla, was statistically significantly associated with inadvertent cannulation of the pancreatic duct in univariate analysis (p<0.001). In contrast, diagnosis of bile duct stones (odds ratio 0.607 (0.401–0.919); p = 0.018) was associated with lower risk of inadvertent cannulation of the pancreatic duct. All data are shown in Table 3.

**Table 3:** Risk factors for inadvertent cannulation of pancreatic duct.

Risk factor	p-value	Odds ratio (CI 95%)
Female sex	0.591	1.117 (0.746–1.673)
Age	0.859	Correlation coefficient -0.009
Age<40	0.791	0.888 (0.367–2.146)
Body weight, Kg	0.183	Correlation coefficient 0.076
Body height, cm	0.678	Correlation coefficient 0.024
BMI	0.238	Correlation coefficient 0.068
BMI ≥ 25	0.078	1.525 (0.952–2.441)
Previous pancreatitis	0.296	0.546 (0.173–1.726)
Chronic pancreatitis	0.769	0.833 (0.247–2.816)
Status post cholecystectomy	0.731	0.912 (0.538–1.546)
Juxtapapillary diverticulum	0.771	1.093 (0.599–1.997)
Serum bilirubin	0.098	Correlation coefficient 0.086
Serum bilirubin <1.3 mg/dL	0.131	0.700 (0.441–1.113)
Bile duct diameter	0.654	Correlation coefficient 0.025
Bile duct diameter <8 mm	0.313	0.783 (0.486–1.260)
ERCP on call	0.193	1.966 (0.698–5.535)
Urgent indication of ERCP	0.679	0.876 (0.468–1.639)
Difficult intubation	<0.001*	7.307 (4.593–11.625)
Diagnosis bile duct stricture	0.362	1.211 (0.802–1.828)
Diagnosis bile duct stones	0.018*	0.607 (0.401–0.919)

For dichotomous data, a chi-square test was performed. Point biserial correlations were used continuous data. P-values < 0.05 are considered statistically significant and are indicated by *. CI, confidence interval; BMI, body mass index; ERCP, endoscopic retrograde cholangiopancreatography.

### Risk factors for difficult cannulation

The rate of difficult cannulation was 49.0% (198/404). There were no statistically significant risk factors for difficult intubation of the papilla duodeni major. All data are presented in Table 4.

**Table 4:** Risk factors for difficult intubation of the papilla duodeni major.

Risk factor	p-value	Odds ratio (CI 95%)
Female sex	0.782	1.057 (0.715–1.562)
Age	0.469	Correlation coefficient 0.036
Age<40	0.160	0.536 (0.222–1.294)
Body weight, Kg	0.980	Correlation coefficient 0.001
Body height, cm	0.950	Correlation coefficient 0.004
BMI	0.943	Correlation coefficient 0.004
BMI ≥ 25	0.332	1.250 (0.796–1.964)
Previous pancreatitis	0.271	1.773 (0.632–4.974)
Chronic pancreatitis	0.512	1.473 (0.460–4.721)
Status post cholecystectomy	0.405	1.241 (0.747–2.062)
Juxtapapillary diverticulum	0.369	0.763 (0.422–1.379)
Serum bilirubin	0.047*	Correlation coefficient 0.102
Serum bilirubin <1.3 mg/dL	0.193	0.746 (0.480–1.160)
Bile duct diameter	0.052	Correlation coefficient 0.106
Bile duct diameter <8 mm	0.023*	0.587 (0.370–0.930)
ERCP on call	0.733	1.197 (0.426–3.365)
Urgent indication for ERCP	0.539	0.829 (0.455–1.510)
Diagnosis bile duct stricture	0.533	1.136 (0.761–1.695)
Diagnosis bile duct stones	0.018*	0.620 (0.417–0.923)

For dichotomous data, a chi-square test was performed. Point biserial correlations were used continuous data. P-values < 0.05 are considered statistically significant and are indicated by *. CI, confidence interval; BMI, body mass index; ERCP, endoscopic retrograde cholangiopancreatography.

### Risk factors for PEP in patients without pancreatic stent and/or diclofenac

15.3% of the patients (62/404) underwent measurements for preventing PEP. In 54 patients, a stent (5 French, various length) was put into the pancreatic duct, 11 patients received diclofenac (100 mg rectally), and 3 of these patients received both. After exclusion of these patients, subgroup analyses showed only inadvertent cannulation of the pancreatic duct as a risk factor for PEP (odds ratio 4.824 (2.221–10.478); p<0.001; Table 5).

**Table 5:** Risk factors for PEP in patients who did not receive PEP prophylaxis.

Risk factor	p-value	Odds ratio (CI 95%)
Female sex	0.643	0.837 (0.393–1.781)
Chronic pancreatitis	0.889	1.162 (0.142–9.488)
Previous pancreatitis	0.236	0.955 (0.932–0.978)
Serum bilirubin <1.3 mg/dL	0.953	1.025 (0.447–2.354)
Bile duct diameter <8 mm	0.692	0.817 (0.301–2.220)
Difficult intubation (<5 attempts)	0.914	1.043 (0.490–2.221
Inadvertent cannulation of pancreatic duct	<0.001*	4.824 (2.221–10.478)

For dichotomous data, a chi-square test was performed. P-values < 0.05 are considered statistically significant and are indicated by *. CI, confidence interval.

## Discussion

This study on risk factors for PEP revealed inadvertent cannulation of the pancreatic duct as an independent risk factor. These date are mostly in line with current studies and guidelines [17, 19].

There are contradicting data on a non-dilated common bile duct as a risk factor for PEP [17, 20]. Our data are conclusive with a metaanalysis by Freeman et al. which did not show an increased risk by multivariate analysis. Sphincter oddi dysfunction, another previously described risk factor, was not examined in our patients [4].

Because most of the risk factors such as sex, age, bilirubin level, or bile duct diameter are not influenceable, efforts must be undertaken to minimize the procedure related risk factors and to optimize prophylactic measurements.

Inadvertent cannulation of the pancreatic duct was associated with an increased risk of PEP (odds ratio 7.468) in our study and others [17]. Procedures associated with cannulation of the pancreatic duct such as pre-cut papillotomy and injection of contrast agent into the pancreatic duct have also been confirmed as risk factors in other studies [5–7, 9, 10]. However, the reasons for inadvertent cannulation of the pancreatic duct are unclear. Our data show that difficult intubation of the papilla, which means more than 5 attempts, is a statistically significant risk factor for inadvertent cannulation. This seems to happen more frequently during ERCPs on call. Although the reasons for that can be manifold, it is conceivable that ERCPs on call are likely more challenging or performed under difficult conditions.

## Conclusion

Inadvertent cannulation of the pancreatic duct is a procedure related risk factor for PEP. Measurements on preventing inadvertent cannulation of the pancreatic duct should be established and studies on prophylactic measurements should focus particularly on patients with inadvertent cannulation of the pancreatic duct.

## Supporting information

S1 File PEP-Risk_Data.xlsx. (XLSX)
