# Correction: Identification of risk factors for post-endoscopic retrograde cholangiopancreatography pancreatitis in a high volume center

**DOI:** 10.1371/journal.pone.0261576

**Published:** 2021-12-14

**Authors:** Veit Phillip, Miriam Schwab, David Haf, Hana Algül

## Notice of Republication

After publication of this article [1], the authors noted that 60 participants who met the inclusion criteria for this study had not been included because of their involvement in a prospective trial. The data were re-analyzed to include these participants as per the study protocol; this affects all five results tables, as well as statements made in the Abstract, Results, and Discussion. A revised version of the article was republished on October 27, 2021, to address this issue. The updated article includes revised versions of Tables 1–5 as well as text updates to the Abstract, Patients and Methods, Results, and Discussion sections. S1 File of [1] has also been updated to include underlying data for all participants included in the reanalyses (n = 404). Please download this article again to view the correct version.

In Table 2 of the republished article, the values reported for ‘Previous pancreatitis’ are incorrect: the correct p-value is 0.182 and the correct odds ratio (CI 95%) is 0.899 (0.870–0.930). In addition, the final 2 sentences of the Discussion (“This seems to happen more frequently…difficult conditions.”) were included in error in the republished article and should be disregarded.

The updated results have been reviewed by two reviewers and a member of *PLOS ONE*’s Editorial Board, who confirmed that the main conclusions of the article are upheld based on the updated dataset and analyses. However, some results statements are no longer supported. Specifically, age is not statistically significantly associated with PEP in univariate analyses, and chronic pancreatitis in the patients’ medical history is not a risk factor for PEP in multivariable analyses. The only risk factor for PEP in multivariate binary logistic regression analysis is inadvertent cannulation of the pancreatic duct (odds ratio 7.468 (2.792–19.975); p<0.001). Non-dilated extrahepatic bile duct is not a statistically significant risk factor for inadvertent cannulation of the pancreatic duct.

For reference, the original article PDF and the underlying data table (n = 344) that was published as S1 File with the original article are provided in S1 and S2 Files of this notice. S3 File includes a track changes version of the manuscript that shows all changes made between the original and updated versions of the article.

The authors apologize for the errors in the original publication. *PLOS ONE* thanks the authors for notifying us of this issue in 2017, and apologizes for our delay in publishing the corrected version.

## Supporting information

S1 File Original article PDF. (PDF)

S2 File PEP-Risk Data. The underlying data table (n = 344) that was published as S1 File with the original version of the article.
(XLSX)

S3 File Track changes version of the manuscript showing changes made between the original and updated publications. (DOC)
