# Supplementary material for: Correction: Identification of risk factors for post-endoscopic retrograde cholangiopancreatography pancreatitis in a high volume center
Source: PLoS One. 2021 Dec 14;16(12):e0261576. doi: 10.1371/journal.pone.0261576 (PMC8670693; doi:10.1371/journal.pone.0261576)
Supplement: S1 File — (PDF) [file pone.0261576.s001.pdf]

PLOS ONE

RESEARCH ARTICLE

Identification of risk factors for post-endoscopic retrograde cholangiopancreatography pancreatitis in a high volume center

Veit Phillip, Miriam Schwab, David Haf, Hana Algül*

Klinik und Poliklinik für Innere Medizin II, Klinikum rechts der Isar, Technische Universität München, München, Germany

* hana.alguel@mri.tum.de

Check for updates

OPEN ACCESS

Citation: Phillip V, Schwab M, Haf D, Algül H (2017) Identification of risk factors for post-endoscopic retrograde cholangiopancreatography pancreatitis in a high volume center. PLoS ONE 12(5): e0177874. https://doi.org/10.1371/journal.pone.0177874

Editor: Zoltán Rakonczay, Jr., University of Szeged, HUNGARY

Received: October 29, 2016

Accepted: May 4, 2017

Published: May 17, 2017

Copyright: © 2017 Phillip et al. This is an open access article distributed under the terms of the Creative Commons Attribution License, which permits unrestricted use, distribution, and reproduction in any medium, provided the original author and source are credited.

Data Availability Statement: All relevant data are within the paper and its Supporting Information files.

Funding: The authors received no specific funding for this work.

Competing interests: The authors have declared that no competing interests exist.

Abstract

Background/Objectives

Pancreatitis is the most common complication of endoscopic retrograde cholangiopancreatography (ERCP). Several patients´ or procedure related risk factors for post-ERCP pancreatitis (PEP) have been suggested. The aim of this study was to validate the risk factors for PEP in a high-volume center.

Methods

All patients undergoing first time ERCP at a tertiary referral center between December 2010 and October 2013 were retrospectively included. PEP was defined according to the Atlanta Classification.

Results

344 patients were included in the final analysis. The risk to develop PEP was increased in patients with chronic pancreatitis (odds ratio 3.7) and after inadvertent cannulation of the pancreatic duct (odds ratio 2.2), which occurred in 26.5% of the patients. Inadvertent cannulation occurred significantly more frequently in patients with difficult cannulation of the papilla duodeni major (odds ratio 12.7; p<0.001). ERCP on call was associated with an increased risk for difficult cannulation (odds ratio 3.0).

Conclusion

Inadvertent cannulation of the pancreatic duct is a procedure related risk factor for PEP. Measurements on preventing inadvertent cannulation of the pancreatic duct should be established and studies on prophylactic measurements should focus particularly on patients with inadvertent cannulation of the pancreatic duct.

Introduction

Endoscopic retrograde cholangiopancreatography (ERCP) is a well-established therapeutic procedure for benign and malignant diseases of the biliopancreatic system. However, post-ERCP pancreatitis (PEP) is the most frequent complication of this procedure with an incidence of about 3–4% in unselected patients and up to 15% in high-risk patients [1–3]. Although most of the cases take a mild or moderate course, about 10% of all patients with PEP develop severe pancreatitis with an estimated pancreatitis-related mortality rate of 3% [1].

Over the last decades, several risk factors for PEP have been suggested. Female sex, young age, non dilated common bile duct, sphincter oddi dysfunction and previous incidence of pancreatitis are considered to be patient related risk factors [4–8]. Furthermore, difficult cannulation of the papilla and procedures associated with cannulation of the pancreatic duct such as pre-cut papillotomy and injection of contrast agent into the pancreatic duct seem to be procedure related risk factors [5–7, 9, 10].

Generally, mechanical trauma of the papilla during ERCP can lead to an edema or spasm of the sphincter of oddi and subsequently restrain the outlet of pancreatic juice thereby causing an elevation of the intrapancreatic pressure. As a consequence, pancreatic secretion is forced to the surrounding pancreas parenchyma, causing autodigestion, a critical event in the pathophysiology of acute pancreatitis [11, 12]. Moreover, cannulation of the pancreatic duct can lead to a damage of the epithelium [13]. In addition to the mechanical and hydrostatic damage, chemical, enzymatic, microbiological, allergic, and thermal factors can further favor PEP [14, 15].

The aim of this study was to validate the supposed and to possibly identify new risk factors for PEP in a high volume tertiary referral center.

Patients and methods

All patients undergoing first time ERCP at the department of endoscopy between December 2010 and October 2013 were included in the study. The endoscopic database, medical charts and laboratory data were analyzed retrospectively.

First time ERCP was performed in 403 patients. 37 patients were excluded due to intended cannulation of the pancreatic duct for pancreatolithiasis, stenosis of the pancreatic duct, or suspected IPMN (intraductal papillary mucinous neoplasm). Further, 22 patients who suffered from acute pancreatitis at the time of ERCP were also excluded. This resulted in 344 patients which were included into the final analysis. The study was conducted in accordance with the Declaration of Helsinki and was approved by the local ethics committee (Ethikkommission der Medizinischen Fakultät der Technischen Universität München, project number 321/14). The ethics committee waived the need for written informed consent for this retrospective study. The number of ERCPs during the 35-month study period was 1425 in total which were performed by twelve endoscopists. Therefore, the average number of yearly ERCP per endoscopist was about 40.

Statistical analyses were performed using IBM SPSS Statistics 23 (SPSS Inc., Chicago, Illinois, USA). For descriptive data, mean ± standard deviation was used for normally distributed data and median (range; interquartile range, IQR) for not normally distributed data. For explorative data analysis, a chi-square test was used to calculate the odds ratio or spearman's correlation to evaluate a statistical relation of suspected risk factors and PEP, inadvertent cannulation of the pancreatic duct, and difficult cannulation of the papilla duodeni major, respectively. P-values < 0.05 were considered to be statistically significant.

Levels of difficulty for cannulation of the papilla duodeni major were categorized as easy (1–5 attempts) and difficult (>5 attempts) cannulation. PEP was defined according to the Atlanta Classification [16].

Results

In total, 344 patients were included in the final analysis. Patients´ characteristics are shown in Table 1.

Incidence of PEP

PEP occurred in 6.1% (21/344) of all patients. All cases were classified as mild pancreatitis.

Incidence of PEP in patients with intended cannulation of the pancreatic duct was 16.2% (6/37).

Risk factors for PEP

In univariate analyses, only age was statistically significantly associated with PEP. However, the correlation was weak (r = 0.107). All data are presented in Table 2.

Multivariate binary logistic regression analysis including the factors age, chronic pancreatitis in the medical history, normal serum bilirubin, non-dilated extrahepatic bile duct, cannulation of the pancreatic duct, and difficult cannulation of the papilla confirmed only chronic pancreatitis in the patients' medical history (p = 0.045) as an independent risk factor for PEP [17, 18]. All 11 patients diagnosed with chronic pancreatitis had an early stage of the disease. The median time period from first diagnosis of chronic pancreatitis to first time ERCP was 5.5 month (IQR, 0–9 month; range 0–72 month). 8 out of 11 patients (73%) had no exocrine or endocrine insufficiency.

Inadvertent cannulation of the pancreatic duct

Inadvertent cannulation of the pancreatic duct occurred in 91/344 (26.5%) of the patients. The risk to develop PEP was increased after inadvertent cannulation of the pancreatic duct (odds ratio 2.204; 95% CI 0.896 to 5.421), however, this was statistically not significant (p = 0.079).

Risk factors for inadvertent cannulation of the pancreatic duct

Difficult cannulation of the papilla duodeni major, which means more than 5 attempts to intubate the papilla, was statistically significantly associated with inadvertent cannulation of the pancreatic duct in univariate analysis ($p<0.001$). In contrast, diagnosis of bile duct stones

Table 1. Patients´ characteristics.

Sex, female (n = 344)	163/344 (47.2%)
Age (n = 344)	67.0 (17.0–94.0;55.0–75.0)
Body weight, kg (n = 251)	73.0 (36.5–185.0; 61.0–83.0)
Body height, cm (n = 248)	171±10
BMI, kg/m2 (n = 245)	24.8 (15.6–64.0;21.8–28.1)
**Indication for ERC** (n = 344)	
Bile duct stones	142 (41.3%)
Jaundice of unknown origin	100 (29.1%)
malignant bile duct stricture	61 (17.7%)
unknown bile duct stricture	14 (4.1%)
benign bile duct stricture	10 (2.9%)
others	17 (4.9%)

Data are presented as number (%), mean ± standard deviation, or median (range; IQR) as applicable. ERC, endoscopic retrograde cholangiography.

https://doi.org/10.1371/journal.pone.0177874.t001

Table 2. Risk factors for post-ERCP pancreatitis.

Risk factor	p-value	odds ratio (CI 95%)
Female sex	0.355	1.519 (0.623–3.704)
Age	0.047*	Correlation coefficient 0.107
Age < 40	0.832	0.800 (0.102–6.284)
Body weight, Kg	0.630	
Body height, cm	0.907	
BMI kg/m^2	0.841	
BMI $\geq$ 25 kg/m^2	0.601	0.753 (0.260–2.184)
Previous pancreatitis	0.602	1.744 (0.210–14.459)
Chronic pancreatitis	0.089	3.673 (0.741–18.201)
Status post cholecystectomy	0.719	0.795 (0.226–2.790)
Juxtapapillary diverticulum	0.730	1.250 (0.352–4.443)
Serum bilirubin <1.3 mg/dL	0.370	1.561 (0.586–4.161)
Bile duct diameter < 8 mm	0.677	0.796 (0.272–2.331)
ERCP on call	0.868	1.192 (0.148–9.578)
Difficult cannulation of papilla	0.559	1.301 (0.537–3.148)
Inadvertent cannulation of the pancreatic duct	0.079	2.204 (0.896–5.421)
Transpancreatic papillotomy	0.695	1.288 (0.362–4.584)
Needle-knife papillotomy	0.743	1.418 (0.174–11.540)
Incomplete bile stone removal	0.478	0.475 (0.058–3.870)
Diagnosis bile duct stones	0.187	1.810 (0.742–4.417)
Diagnosis bile duct stricture	0.181	0.502 (0.179–1.403)
Prophylactic use of Diclofenac	0.674	1.565 (0.191–12.841)
Pancreatic duct stent	0.229	2.174 (0.596–7.927

For dichotomous data, a chi-square test was performed. Pearson and Spearman correlations were used for normally and not-normally distributed intervals, respectively. P-values < 0.05 are considered statistically significant and are indicated by *. CI, confidence interval; BMI, body mass index; ERCP, endoscopic retrograde cholangiopancreatography.

https://doi.org/10.1371/journal.pone.0177874.t002

(odds ratio 0.485 (0.291–0.807; p = 0.005) and non-dilated extrahepatic bile duct (odds ratio 0.539 (0.292–0.995); p = 0.046) were associated with lower risk of inadvertent cannulation of the pancreatic duct. All data are shown in Table 3.

Risk factors for difficult cannulation

The rate of difficult cannulation was 46.2% (159/344). ERCP on call (performed outside the operational hours) was associated with an increased risk of difficult cannulation (>5 attempts) of the papilla duodeni major (odd ratio 3.037 (0.934–9.880), however, this increased risk did not reach statistical significance (p = 0.053). All data are presented in Table 4. Multiple regression analysis including the factors with an odds ratio >1 showed no statistically significant risk factor for difficult intubation of the papilla duodeni major.

Risk factors for PEP in patients without pancreatic stent and/or diclofenac

9.9% of the patients (34/344) underwent measurements for preventing PEP. In 26 patients, a stent (5 French, various length) was put into the pancreatic duct, 11 patients received diclofenac (100 mg rectally), and 3 of these patients received both. After exclusion of these patients,

Table 3. Risk factors for inadvertent cannulation of pancreatic duct.

Risk factor	p-value	Odds ratio (CI 95%)
Female sex	0.445	0.829 (0.512–1.342)
Age	0.585	
Age<40	0.879	0.922 (0.325–2.614)
Body weight, Kg	0.585	
Body height, cm	0.763	
BMI	0.496	
BMI $\geq$ 25	0.722	1.109 (0.627–1.963)
Previous pancreatitis	0.796	1.198 (0.303–4.735)
Chronic pancreatitis	0.527	0.609 (0.129–2.874)
Status post cholecystectomy	0.135	0.587 (0.290–1.188)
Juxtapapillary diverticulum	0.750	0.884 (0.415–1.884)
Serum bilirubin	0.035*	Correlation coefficient 0.119
Serum bilirubin <1.3 mg/dL	0.180	0.676 (0.381–1.200)
Bile duct diameter	0.022*	Correlation coefficient 0.137
Bile duct diameter <8 mm	0.046*	0.539 (0.292–0.995)
ERCP on call	0.423	1.576 (0.514–4.833)
Urgent indication of ERCP	0.890	0.950 (0.457–1.973)
Difficult cannulation	<0.001*	12.741 (6.692–24.255)
Diagnosis bile duct stricture	0.825	1.057 (0.646–1.731)
Diagnosis bile duct stones	0.005*	0.485 (0.291–0.807)

For dichotomous data, a chi-square test was performed. Pearson and Spearman correlations were used for normally and not-normally distributed intervals, respectively. P-values < 0.05 are considered statistically significant and are indicated by *. CI, confidence interval; BMI, body mass index; ERCP, endoscopic retrograde cholangiopancreatography.

https://doi.org/10.1371/journal.pone.0177874.t003

subgroup analyses showed an odds ratio close to or greater than 2 regarding PEP for chronic pancreatitis or previous pancreatitis in the medical history, normal serum bilirubin, and inadvertent cannulation of the pancreatic duct (Table 5). However, in multiple regression analysis including these factors, only patients with chronic pancreatitis showed a statistically significant risk to develop PEP (p = 0.045).

Discussion

This study on risk factors for PEP revealed chronic pancreatitis as an independent risk factor. Furthermore, females as well as patients with normal serum bilirubin and previous pancreatitis seem to define the high-risk patient cohort. These date are mostly in line with current studies and guidelines [17–20]. While a previous study showed a protective effect of chronic pancreatitis, our data showed an increased risk of PEP in patients with chronic pancreatitis [17, 21]. The reason for this effect is not known. It is very likely that the extent of chronic pancreatitis has a significant influence on its relevance as a risk factor for PEP. While a fibrotic gland with the loss of the exocrine compartment is potentially less susceptible to PEP, CP with ongoing inflammation in affected acinar cells is more prone to PEP. And yet, this is speculative, as in none of these studies the incidence of PEP has been evaluated in relation to the stadium (e.g. according to the Cambridge classification) of CP [22]. However, the patients in our cohort had rather early stage of chronic pancreatitis.

There are contradicting data on a non-dilated common bile duct as a risk factor for PEP [17, 20]. Our data are conclusive with a metaanalysis by Freeman et al. which did not show an

Table 4. Risk factors for difficult intubation of the papilla duodeni major.

Risk factor	p-value	Odds ratio (CI 95%)
Female sex	0.941	0.984 (0.644–1.505
Age	0.982	
Age<40	0.134	0.479 (0.180–1.277)
Body weight, Kg	0.778	
Body height, cm	0.351	
BMI	0.247	
BMI $\geq$ 25	0.255	0.746 (0.451–1.236)
Previous pancreatitis	0.808	1.169 (0.332–4.113)
Chronic pancreatitis	0.505	0.656 (0.189–2.284)
Status post cholecystectomy	0.348	0.762 (0.431–1.346)
Juxtapapillary diverticulum	0.726	1.124 (0.585–2.158)
Serum bilirubin	0.148	
Serum bilirubin <1.3 mg/dL	0.533	0.856 (0.526–1.395)
Bile duct diameter	0.069	
Bile duct diameter <8 mm	0.283	0.760 (0.460–1.255)
ERCP on call	0.053	3.037 (0.934–9.880)
Urgent indication for ERCP	0.487	1.254 (0.662–2.377)
Diagnosis bile duct stricture	0.759	1.071 (0.691–1.659)

For dichotomous data, a chi-square test was performed. Pearson and Spearman correlations were used for normally and not-normally distributed intervals, respectively. P-values < 0.05 are considered statistically significant and are indicated by *. CI, confidence interval; BMI, body mass index; ERCP, endoscopic retrograde cholangiopancreatography.

https://doi.org/10.1371/journal.pone.0177874.t004

increased risk by multivariate analysis. Sphincter oddi dysfunction, another previously described risk factor, was not examined in our patients [4].

Because most of the risk factors such as sex, age, bilirubin level, or bile duct diameter are not influenceable, efforts must be undertaken to minimize the procedure related risk factors and to optimize prophylactic measurements.

Inadvertent cannulation of the pancreatic duct was associated with an increased risk of PEP (odds ration 2.2) in our study and others [17]. Procedures associated with cannulation of the pancreatic duct such as pre-cut papillotomy and injection of contrast agent into the pancreatic duct have also been confirmed as risk factors in other studies [5–7, 9, 10]. However, the reasons for inadvertent cannulation of the pancreatic duct are unclear. Our data show that difficult intubation of the papilla, which means more than 5 attempts, is a statistically significant risk factor for inadvertent cannulation. This seems to happen more frequently during ERCPs

Table 5. Risk factors for PEP in patients who did not receive PEP prophylaxis.

Risk factor	p-value	Odds ratio (CI 95%)
Chronic pancreatitis	0.060	4.207 (0.834–21.213)
Previous pancreatitis	0.524	1.972 (0.235–16.539)
Serum bilirubin <1.3 mg/dL	0.303	1.763 (0.592–5.244)
Inadvertent cannulation of pancreatic duct	0.280	1.803 (0.610–5.329)

For dichotomous data, a chi-square test was performed. P-values < 0.05 are considered statistically significant and are indicated by *. CI, confidence interval.

https://doi.org/10.1371/journal.pone.0177874.t005

on call. Although the reasons for that can be manifold, it is conceivable that ERCPs on call are likely more challenging or performed under difficult conditions.

Conclusion

Inadvertent cannulation of the pancreatic duct is a procedure related risk factor for PEP. Measurements on preventing inadvertent cannulation of the pancreatic duct should be established and studies on prophylactic measurements should focus particularly on patients with inadvertent cannulation of the pancreatic duct.

Supporting information

S1 File. PEP-Risk_Data.xlsx.
(XLSX)

Author Contributions

Conceptualization: VP MS DH HA.

Data curation: MS DH.

Formal analysis: VP.

Funding acquisition: VP MS DH HA.

Investigation: VP MS DH HA.

Methodology: VP HA.

Project administration: VP HA.

Resources: MS DH.

Supervision: HA.

Validation: VP HA.

Visualization: VP MS DH HA.

Writing – original draft: VP.

Writing – review & editing: VP MS DH HA.

References

1. Andriulli A, Loperfido S, Napolitano G, Niro G, Valvano MR, Spirito F, et al. Incidence rates of post-ERCP complications: a systematic survey of prospective studies. Am J Gastroenterol. 2007; 102(8):1781–8. https://doi.org/10.1111/j.1572-0241.2007.01279.x PMID: 17509029
2. Kapral C, Muhlberger A, Wewalka F, Duller C, Knoflach P, Schreiber F, et al. Quality assessment of endoscopic retrograde cholangiopancreatography: results of a running nationwide Austrian benchmarking project after 5 years of implementation. Eur J Gastroenterol Hepatol. 2012; 24(12):1447–54. https://doi.org/10.1097/MEG.0b013e3283583c6f PMID: 23114747
3. Kochar B, Akshintala VS, Afghani E, Elmunzer BJ, Kim KJ, Lennon AM, et al. Incidence, severity, and mortality of post-ERCP pancreatitis: a systematic review by using randomized, controlled trials. Gastrointest Endosc. 2015; 81(1):143–9 e9. https://doi.org/10.1016/j.gie.2014.06.045 PMID: 25088919
4. Chen YK, Foliente RL, Santoro MJ, Walter MH, Collen MJ. Endoscopic sphincterotomy-induced pancreatitis: increased risk associated with nondilated bile ducts and sphincter of Oddi dysfunction. Am J Gastroenterol. 1994; 89(3):327–33. PMID: 8122639

5. Loperfido S, Angelini G, Benedetti G, Chilovi F, Costan F, De Berardinis F, et al. Major early complications from diagnostic and therapeutic ERCP: a prospective multicenter study. Gastrointest Endosc. 1998; 48(1):1–10. PMID: 9684657
6. Dickinson RJ, Davies S. Post-ERCP pancreatitis and hyperamylasaemia: the role of operative and patient factors. Eur J Gastroenterol Hepatol. 1998; 10(5):423–8. PMID: 9619390
7. Masci E, Mariani A, Curioni S, Testoni PA. Risk factors for pancreatitis following endoscopic retrograde cholangiopancreatography: a meta-analysis. Endoscopy. 2003; 35(10):830–4. https://doi.org/10.1055/s-2003-42614 PMID: 14551860
8. Sherman S, Ruffolo TA, Hawes RH, Lehman GA. Complications of endoscopic sphincterotomy. A prospective series with emphasis on the increased risk associated with sphincter of Oddi dysfunction and nondilated bile ducts. Gastroenterology. 1991; 101(4):1068–75. PMID: 1889699
9. Freeman ML, Nelson DB, Sherman S, Haber GB, Herman ME, Dorsher PJ, et al. Complications of endoscopic biliary sphincterotomy. N Engl J Med. 1996; 335(13):909–18. https://doi.org/10.1056/NEJM199609263351301 PMID: 8782497
10. Johnson GK, Geenen JE, Johanson JF, Sherman S, Hogan WJ, Cass O. Evaluation of post-ERCP pancreatitis: potential causes noted during controlled study of differing contrast media. Midwest Pancreaticobiliary Study Group. Gastrointest Endosc. 1997; 46(3):217–22. PMID: 9378207
11. Sherman S, Lehman GA. ERCP- and endoscopic sphincterotomy-induced pancreatitis. Pancreas. 1991; 6(3):350–67. PMID: 1713676
12. Lesina M, Wormann SM, Neuhofer P, Song L, Algul H. Interleukin-6 in inflammatory and malignant diseases of the pancreas. Semin Immunol. 2014; 26(1):80–7. https://doi.org/10.1016/j.smim.2014.01.002 PMID: 24572992
13. Bub H, Burner W, Riemann JF, Stolte M. Morphology of the pancreatic ductal epithelium after traumatization of the papilla of Vater or endoscopic retrograde pancreatography with various contrast media in cats. Scand J Gastroenterol. 1983; 18(5):581–92. PMID: 6675180
14. Testoni PA. Why the incidence of post-ERCP pancreatitis varies considerably? Factors affecting the diagnosis and the incidence of this complication. JOP. 2002; 3(6):195–201. PMID: 12432186
15. D. B. The etiology of post-ERCP pancreatitis. Gastrointestinal Endoscopy. 1989; 35:189A.
16. Bradley EL 3rd. A clinically based classification system for acute pancreatitis. Summary of the International Symposium on Acute Pancreatitis, Atlanta, Ga, September 11 through 13, 1992. Arch Surg. 1993; 128(5):586–90. Epub 1993/05/01. PMID: 8489394
17. Dumonceau JM, Andriulli A, Elmunzer BJ, Mariani A, Meister T, Deviere J, et al. Prophylaxis of post-ERCP pancreatitis: European Society of Gastrointestinal Endoscopy (ESGE) Guideline—updated June 2014. Endoscopy. 2014; 46(9):799–815. https://doi.org/10.1055/s-0034-1377875 PMID: 25148137
18. Committee ASoP, Anderson MA, Fisher L, Jain R, Evans JA, Appalaneni V, et al. Complications of ERCP. Gastrointest Endosc. 2012; 75(3):467–73. https://doi.org/10.1016/j.gie.2011.07.010 PMID: 22341094
19. Thiruvengadam NR, Forde KA, Ma GK, Ahmad N, Chandrasekhara V, Ginsberg GG, et al. Rectal Indomethacin Reduces Pancreatitis in High- and Low-Risk Patients Undergoing Endoscopic Retrograde Cholangiopancreatography. Gastroenterology. 2016; 151(2):288–97 e4. https://doi.org/10.1053/j.gastro.2016.04.048 PMID: 27215656
20. Freeman ML. Pancreatic stents for prevention of post-endoscopic retrograde cholangiopancreatography pancreatitis. Clin Gastroenterol Hepatol. 2007; 5(11):1354–65. https://doi.org/10.1016/j.cgh.2007.09.007 PMID: 17981248
21. Freeman ML, DiSario JA, Nelson DB, Fennerty MB, Lee JG, Bjorkman DJ, et al. Risk factors for post-ERCP pancreatitis: a prospective, multicenter study. Gastrointest Endosc. 2001; 54(4):425–34. PMID: 11577302
22. Zhao ZH, Hu LH, Ren HB, Zhao AJ, Qian YY, Sun XT, et al. Incidence and Risk Factors for post-ERCP Pancreatitis in Chronic Pancreatitis. Gastrointest Endosc. 2017.
