# Supplementary material for: Correction: Identification of risk factors for post-endoscopic retrograde cholangiopancreatography pancreatitis in a high volume center
Source: PLoS One. 2021 Dec 14;16(12):e0261576. doi: 10.1371/journal.pone.0261576 (PMC8670693; doi:10.1371/journal.pone.0261576)
Supplement: S3 File — (DOC) [file pone.0261576.s003.doc]

Identification of risk factors for post-endoscopic retrograde cholangiopancreatography pancreatitis in a high volume center

Veit Phillip, Miriam Schwab, David Haf, Hana Algül*

Klinik und Poliklinik für Innere Medizin II, Klinikum rechts der Isar, Technische Universität München, München, Germany

* Corresponding author

E-mail: hana.alguel@mri.tum.de (HA)

The authors declare no competing financial interests.

**Abstract**

Background/Objectives: Pancreatitis is the most common complication of endoscopic retrograde cholangiopancreatography (ERCP). Several patients´ or procedure related risk factors for post-ERCP pancreatitis (PEP) have been suggested. The aim of this study was to validate the risk factors for PEP in a high-volume center.

Methods: All patients undergoing first time ERCP at a tertiary referral center between December 2010 and October 2013 were retrospectively included. PEP was defined according to the Atlanta Classification.

Results: ~~344~~ 404 patients were included in the final analysis. The risk to develop PEP was increased in patients ~~with chronic pancreatitis (odds ratio 3.7) and~~ after inadvertent cannulation of the pancreatic duct (~~odds ratio 2.2~~ odds ratio 7.468 (2.792-19.975); p<0.001), which occurred in ~~26.5 %~~ 37.4 % of the patients. Inadvertent cannulation occurred significantly more frequently in patients with difficult intubation of the papilla duodeni major (odds ratio ~~12.7~~ 7.3; p<0.001). ~~ERCP on call was associated with an increased risk for difficult intubation (odds ratio 3.0).~~

Conclusion: Inadvertent cannulation of the pancreatic duct is a procedure related risk factor for PEP. Measurements on preventing inadvertent cannulation of the pancreatic duct should be established and studies on prophylactic measurements should focus particularly on patients with inadvertent cannulation of the pancreatic duct.

**Introduction**

Endoscopic retrograde cholangiopancreatography (ERCP) is a well-established therapeutic procedure for benign and malignant diseases of the biliopancreatic system. However, post-ERCP pancreatitis (PEP) is the most frequent complication of this procedure with an incidence of about 3-4% in unselected patients and up to 15% in high-risk patients [1-3]. Although most of the cases take a mild or moderate course, about 10% of all patients with PEP develop severe pancreatitis with an estimated pancreatitis-related mortality rate of 3% [1].

Over the last decades, several risk factors for PEP have been suggested. Female sex, young age, non dilated common bile duct, sphincter oddi dysfunction and previous incidence of pancreatitis are considered to be patient related risk factors [4-8]. Furthermore, difficult intubation of the papilla and procedures associated with cannulation of the pancreatic duct such as pre-cut papillotomy and injection of contrast agent into the pancreatic duct seem to be procedure related risk factors [5-7, 9, 10].

Generally, mechanical trauma of the papilla during ERCP can lead to an edema or spasm of the sphincter of oddi and subsequently restrain the outlet of pancreatic juice thereby causing an elevation of the intrapancreatic pressure. As a consequence, pancreatic secretion is forced to the surrounding pancreas parenchyma, causing autodigestion, a critical event in the pathophysiology of acute pancreatitis [11, 12]. Moreover, cannulation of the pancreatic duct can lead to a damage of the epithelium [13]. In addition to the mechanical and hydrostatic damage, chemical, enzymatic, microbiological, allergic, and thermal factors can further favor PEP [14, 15].

The aim of this study was to validate the supposed and to possibly identify new risk factors for PEP in a high volume tertiary referral center.

**Patients and methods**

All patients undergoing first time ERCP at the department of endoscopy between December 2010 and October 2013 were included in the study. The endoscopic database, medical charts and laboratory data were analyzed retrospectively.

First time ERCP was performed in ~~403~~ 463 patients. 37 patients were excluded due to intended cannulation of the pancreatic duct for pancreatolithiasis, stenosis of the pancreatic duct, or suspected IPMN (intraductal papillary mucinous neoplasm). Further, 22 patients who suffered from acute pancreatitis at the time of ERCP were also excluded. This resulted in ~~344~~ 404 patients which were included into the final analysis. The study was conducted in accordance with the Declaration of Helsinki and was approved by the local ethics committee (Ethikkommission der Medizinischen Fakultät der Technischen Universität München, project number 321/14). The ethics committee waived the need for written informed consent for this retrospective study. The number of ERCPs during the 35-month study period was 1425 in total which were performed by twelve endoscopists. Therefore, the average number of yearly ERCP per endoscopist was about 40.

Statistical analyses were performed using IBM SPSS Statistics 23 (SPSS Inc., Chicago, Illinois, USA). For descriptive data, mean ± standard deviation was used for normally distributed data and median (range; interquartile range, IQR) for not normally distributed data. For explorative data analysis, a chi-square test was used to calculate the odds ratio or spearman’s correlation to evaluate a statistical relation of suspected risk factors and PEP, inadvertent cannulation of the pancreatic duct, and difficult intubation of the papilla duodeni major, respectively. To calculate correlations between continuous risk factors and dichotomous outcome we used a point biserial correlation. P-values < 0.05 were considered to be statistically significant.

Levels of difficulty for intubation of the papilla duodeni major were categorized as easy (1-5 attempts) and difficult (>5 attempts) intubation. PEP was defined according to the Atlanta Classification [16].

**Results**

In total, ~~344~~ 404 patients were included in the final analysis. Patients´ characteristics are shown in table 1.

Table 1: Patients´ characteristics

| Sex, female  n=344  n=404 | 163/344 (47.2 %)  191/404 (47.3 %) |
| --- | --- |
| Age  n=344  n=404 | 67.0 (17.0-94.0;55.0-75.0)  67.0 (17.0-94.0; 56.0-75.0) |
| Body weight, kg  n=251  n=309 | 73.0 (36.5-185.0; 61.0-83.0)  74.0 (36.5-185.0;62.0-84.0) |
| Body height, cm  n=248  n=305 | 171±10  171±10 |
| BMI, kg/m2  n=245  n=302 | 24.8 (15.6-64.0;21.8-28.1)  24.9 (15.6-64.0;21.8-28.4) |
| **Indication for ERC**  n=344  n=404 |  |
| Bile duct stones | 142 (41.3 %)  166 (41.1 %) |
| Jaundice of unknown origin | 100 (29.1 %)  112 (27.7 %) |
| malignant bile duct stricture | 61 (17.7 %)  74 (18.3%) |
| unknown bile duct stricture | 14 (4.1 %)  19 (4.7 %) |
| benign bile duct stricture | 10 (2.9 %)  12 (3.0 %) |
| others | 17 (4.9 %)  21 (5.2 %) |

Data are presented as number (%), mean ± standard deviation, or median (range; IQR) as applicable. ERC, endoscopic retrograde cholangiography.

*Incidence of PEP*

PEP occurred in ~~6.1 % (21/344)~~ 9.7 % (39/404) of all patients. ~~All cases were classified as mild pancreatitis.~~ One case was classified as severe pancreatitis, all others as mild pancreatitis.

Incidence of PEP in patients with intended cannulation of the pancreatic duct was 16.2 % (6/37).

**Risk factors for PEP**

In univariate analyses, only ~~age~~ inadvertent cannulation of the pancreatic duct was statistically significantly associated with PEP (odds ratio 4.373 (2.142-8.927; p<0.001). All data are presented in table 2.

Multivariate binary logistic regression analysis including the factors age, chronic pancreatitis in the medical history, normal serum bilirubin, non-dilated extrahepatic bile duct, cannulation of the pancreatic duct, and difficult intubation of the papilla confirmed ~~only chronic pancreatitis in the patients’ medical history (p=0.045)~~ inadvertent cannulation of the pancreatic duct (odds ratio 7.468 (2.792-19.975); p<0.001) as an independent risk factor for PEP [17, 18].

(All ~~11~~ 12 patients diagnosed with chronic pancreatitis had an early stage of the disease. The median time period from first diagnosis of chronic pancreatitis to first time ERCP was ~~5.5~~ 6.0 month (IQR, ~~0-9~~ 0-60 month; range ~~0-72~~ 0-102 month). ~~8~~ 9 out of ~~11~~ 12 patients (~~73~~ 75 %) had no exocrine or endocrine insufficiency.)

Table 2: Risk factors for post-ERCP pancreatitis

| **Risk factor** | **p-value** | **odds ratio (CI 95%)** |
| --- | --- | --- |
| Female sex | 0.355  0.627 | 1.519 (0.623-3.704)  0.848 (0.436-1.650) |
| Age | 0.047*  0.905 | Correlation coefficient 0.107  Correlation coefficient -0.006 |
| Age < 40 | 0.832  0.571 | 0.800 (0.102-6.284)  1.438 (0.407-5.073) |
| Body weight, Kg | 0.630  0.067 | Correlation coefficient -0.104 |
| Body height, cm | 0.907  0.530 | Correlation coefficient -0.036 |
| BMI kg/m^2^ | 0.841  0.062 | Correlation coefficient -0.107 |
| BMI ≥ 25 kg/m^2^ | 0.601  0.261 | 0.753 (0.260-2.184)  0.638 (0.291-1.402) |
| Previous pancreatitis | 0.602  0.182 | 1.744 (0.210-14.459)  0.899 (0.870-0.930) |
| Chronic pancreatitis | 0.089  0.404 | 3.673 (0.741-18.201)  1.919 (0.405-9.091) |
| Status post cholecystectomy | 0.719  0.676 | 0.795 (0.226-2.790)  1.191 (0.523-2.710) |
| Juxtapapillary diverticulum | 0.730  0.640 | 1.250 (0.352-4.443)  0.773 (0.263-2.274) |
| Serum bilirubin <1.3 mg/dL | 0.370  0.806 | 1.561 (0.586-4.161)  1.095 (0.530-2.264) |
| Bile duct diameter < 8 mm | 0.677  0.678 | 0.796 (0.272-2.331)  0.834 (0.353-1.969) |
| ERCP on call | 0.868  0.690 | 1.192 (0.148-9.578)  0.660 (0.084-5.157) |
| Difficult intubation of papilla | 0.559  0.525 | 1.301 (0.537-3.148)  1.239 (0.639-2.403) |
| Inadvertent cannulation of the pancreatic duct | 0.079  <0.001* | 2.204 (0.896-5.421)  4.373 (2.142-8.927) |
| Transpancreatic papillotomy | 0.695  0.149 | 1.288 (0.362-4.584)  1.753 (0.811-3.787) |
| Needle-knife papillotomy | 0.743  0.746 | 1.418 (0.174-11.540)  0.713 (0.091-5.598) |
| Incomplete bile stone removal | 0.478  0.873 | 0.475 (0.058-3.870)  1.108 (0.318-3.860) |
| Diagnosis bile duct stones | 0.187  0.163 | 1.810 (0.742-4.417)  1.598 (0.824-3.102) |
| Diagnosis bile duct stricture | 0.181  0.456 | 0.502 (0.179-1.403)  0.767 (0.382-1.542) |
| Prophylactic use of Diclofenac | 0.674  0.949 | 1.565 (0.191-12.841)  0.934 (0.116-7.498) |
| Pancreatic duct stent | 0.229  0.168 | 2.174 (0.596-7.927  1.790 (0.775-4.131) |

For dichotomous data, a chi-square test was performed. Point biserial correlations were used continuous data. P-values < 0.05 are considered statistically significant and are indicated by *. CI, confidence interval; BMI, body mass index; ERCP, endoscopic retrograde cholangiopancreatography.

**Risk factors for inadvertent cannulation of the pancreatic duct**

Inadvertent cannulation of the pancreatic duct occurred in ~~91/344 (26.5 %)~~ 151/404 (37.4 %) of the patients. The risk to develop PEP was statistically significantly increased after inadvertent cannulation of the pancreatic duct ~~(odds ratio 2.204; 95% CI 0.896 to 5.421)~~ (odds ratio 4.373; 95% CI 2.142-8.927; p<0.001), ~~however, this was statistically not significant (p=0.079).~~

**~~Risk factors for inadvertent cannulation of the pancreatic duct~~**

Difficult intubation of the papilla duodeni major, which means more than 5 attempts to intubate the papilla, was statistically significantly associated with inadvertent cannulation of the pancreatic duct in univariate analysis (p<0.001). In contrast, diagnosis of bile duct stones ~~(odds ratio 0.485 (0.291-0.807; p=0.005)~~ (odds ratio 0.607 (0.401-0.919; p=0.018) ~~and non-dilated extrahepatic bile duct (odds ratio 0.539 (0.292-0.995); p=0.046) were~~ was associated with lower risk of inadvertent cannulation of the pancreatic duct. All data are shown in table 3.

Table 3: Risk factors for inadvertent cannulation of pancreatic duct

| Risk factor | p-value | Odds ratio (CI 95%) |
| --- | --- | --- |
| Female sex | 0.445  0.591 | 0.829 (0.512-1.342)  1.117 (0.746-1.673) |
| Age | 0.585  0.859 | Correlation coefficient -0.009 |
| Age<40 | 0.879  0.791 | 0.922 (0.325-2.614)  0.888 (0.367-2.146) |
| Body weight, Kg | 0.585  0.183 | Correlation coefficient 0.076 |
| Body height, cm | 0.763  0.678 | Correlation coefficient 0.024 |
| BMI | 0.496  0.238 | Correlation coefficient 0.068 |
| BMI ≥ 25 | 0.722  0.078 | 1.109 (0.627-1.963)  1.525 (0.952-2.441) |
| Previous pancreatitis | 0.796  0.296 | 1.198 (0.303-4.735)  0.546 (0.173-1.726) |
| Chronic pancreatitis | 0.527  0.769 | 0.609 (0.129-2.874)  0.833 (0.247-2.816) |
| Status post cholecystectomy | 0.135  0.731 | 0.587 (0.290-1.188)  0.912 (0.538-1.546) |
| Juxtapapillary diverticulum | 0.750  0.771 | 0.884 (0.415-1.884)  1.093 (0.599-1.997) |
| Serum bilirubin | 0.035*  0.098 | Correlation coefficient 0.119  Correlation coefficient 0.086 |
| Serum bilirubin <1.3 mg/dL | 0.180  0.131 | 0.676 (0.381-1.200)  0.700 (0.441-1.113) |
| Bile duct diameter | 0.022*  0.654 | Correlation coefficient 0.137  Correlation coefficient 0.025 |
| Bile duct diameter <8 mm | 0.046*  0.313 | 0.539 (0.292-0.995)  0.783 (0.486-1.260) |
| ERCP on call | 0.423  0.193 | 1.576 (0.514-4.833)  1.966 (0.698-5.535) |
| Urgent indication of ERCP | 0.890  0.679 | 0.950 (0.457-1.973)  0.876 (0.468-1.639) |
| Difficult intubation | <0.001*  <0.001* | 12.741 (6.692-24.255)  7.307 (4.593-11.625) |
| Diagnosis bile duct stricture | 0.825  0.362 | 1.057 (0.646-1.731)  1.211 (0.802-1.828) |
| Diagnosis bile duct stones | 0.005*  0.018* | 0.485 (0.291-0.807)  0.607 (0.401-0.919) |

For dichotomous data, a chi-square test was performed. Point biserial correlations were used for continuous data. P-values < 0.05 are considered statistically significant and are indicated by *. CI, confidence interval; BMI, body mass index; ERCP, endoscopic retrograde cholangiopancreatography.

**Risk factors for difficult intubation**

The rate of difficult intubation was ~~46.2 % (159/344)~~ 49.0 % (198/404). ~~ERCP on call (performed outside the operational hours) was associated with an increased risk of difficult intubation (>5 attempts) of the papilla duodeni major (odd ratio 3.037 (0.934-9.880), (odds ratio 1.197 (0.426-3.365) however, this increased risk did not reach statistical significance (p=0.053) (p=0.733).~~ There were no statistically significant risk factors for difficult intubation of the papilla duodeni major. All data are presented in table 4. ~~Multiple regression analysis including the factors with an odds ratio >1 showed no statistically significant risk factor for difficult intubation of the papilla duodeni major.~~

Table 4: Risk factors for difficult intubation of the papilla duodeni major

| Risk factor | p-value | Odds ratio (CI 95%) |
| --- | --- | --- |
| Female sex | 0.941  0.782 | 0.984 (0.644-1.505  1.057 (0.715-1.562) |
| Age | 0.982  0.469 | Correlation coefficient 0.036 |
| Age<40 | 0.134  0.160 | 0.479 (0.180-1.277)  0.536 (0.222-1.294) |
| Body weight, Kg | 0.778  0.980 | Correlation coefficient 0.001 |
| Body height, cm | 0.351  0.950 | Correlation coefficient 0.004 |
| BMI | 0.247  0.943 | Correlation coefficient 0.004 |
| BMI ≥ 25 | 0.255  0.332 | 0.746 (0.451-1.236)  1.250 (0.796-1.964) |
| Previous pancreatitis | 0.808  0.271 | 1.169 (0.332-4.113)  1.773 (0.632-4.974) |
| Chronic pancreatitis | 0.505  0.512 | 0.656 (0.189-2.284)  1.473 (0.460-4.721) |
| Status post cholecystectomy | 0.348  0.405 | 0.762 (0.431-1.346)  1.241 (0.747-2.062) |
| Juxtapapillary diverticulum | 0.726  0.369 | 1.124 (0.585-2.158)  0.763 (0.422-1.379) |
| Serum bilirubin | 0.148  0.047* | Correlation coefficient 0.102 |
| Serum bilirubin <1.3 mg/dL | 0.533  0.193 | 0.856 (0.526-1.395)  0.746 (0.480-1.160) |
| Bile duct diameter | 0.069  0.052 | Correlation coefficient 0.106 |
| Bile duct diameter <8 mm | 0.283  0.023* | 0.760 (0.460-1.255)  0.587 (0.370-0.930) |
| ERCP on call | 0.053  0.733 | 3.037 (0.934-9.880)  1.197 (0.426-3.365) |
| Urgent indication for ERCP | 0.487  0.539 | 1.254 (0.662-2.377)  0.829 (0.455-1.510) |
| Diagnosis bile duct stricture | 0.759  0.533 | 1.071 (0.691-1.659)  1.136 (0.761-1.695) |
| Diagnosis bile duct stones | 0.018* | 0.620 (0.417-0.923) |

For dichotomous data, a chi-square test was performed. Point biserial correlations were used for continuous data. P-values < 0.05 are considered statistically significant and are indicated by *. CI, confidence interval; BMI, body mass index; ERCP, endoscopic retrograde cholangiopancreatography.

**Risk factors for PEP in patients without pancreatic stent and/or diclofenac**

~~9.9 %~~ 15.3 % of the patients ~~(34/344)~~ (62/404) underwent measures for preventing PEP. In ~~26~~ 54 patients, a stent (5 French, various length) was put into the pancreatic duct, 11 patients received diclofenac (100 mg rectally), and 3 of these patients received both. After exclusion of these patients, subgroup analyses showed only inadvertent cannulation of the pancreatic duct as a risk factor for PEP (odds ratio 4.824 (2.221-10.478; p<0.001; table 5). ~~an odds ratio close to or greater than 2 regarding PEP for chronic pancreatitis or previous pancreatitis in the medical history, normal serum bilirubin, and inadvertent cannulation of the pancreatic duct (table 5). However, in multiple regression analysis including these factors, only patients with chronic pancreatitis showed a statistically significant risk to develop PEP (p=0.045).~~

Table 5: Risk factors for PEP in patients who did not receive PEP prophylaxis

| **Risk factor** | **p-value** | **Odds ratio (CI 95%)** |
| --- | --- | --- |
| Female sex | 0.643 | 0.837 (0.393-1.781) |
| Chronic pancreatitis | ~~0.060~~  0.889 | ~~4.207 (0.834-21.213)~~  1.162 (0.142-9.488) |
| Previous pancreatitis | ~~0.524~~  0.236 | ~~1.972 (0.235-16.539)~~  0.955 (0.932-0.978) |
| Serum bilirubin <1.3 mg/dL | ~~0.303~~  0.953 | ~~1.763 (0.592-5.244)~~  1.025 (0.447-2.354) |
| Bile duct diameter <8 mm | 0.692 | 0.817 (0.301-2.220) |
| Difficult intubation  (<5 attempts) | 0.914 | 1.043 (0.490-2.221 |
| Inadvertent cannulation of pancreatic duct | ~~0.280~~  <0.001* | ~~1.803 (0.610-5.329)~~  4.824 (2.221-10.478) |

For dichotomous data, a chi-square test was performed. P-values < 0.05 are considered statistically significant and are indicated by *. CI, confidence interval.

**Discussion**

This study on risk factors for PEP revealed ~~chronic pancreatitis~~ inadvertent cannulation of the pancreatic duct as an independent risk factor. ~~Furthermore, females as well as patients with normal serum bilirubin and previous pancreatitis seem to define the high-risk patient cohort~~. These date are mostly in line with current studies and guidelines[17, 19] ~~[17, 18, 20, 21]~~. ~~While a previous study showed a protective effect of chronic pancreatitis, our data showed an increased risk of PEP in patients with chronic pancreatitis [17, 22]. The reason for this effect is not known. It is very likely that the extent of chronic pancreatitis has a significant influence on its relevance as a risk factor for PEP. While a fibrotic gland with the loss of the exocrine compartment is potentially less susceptible to PEP, CP with ongoing inflammation in affected acinar cells is more prone to PEP. And yet, this is speculative, as in none of these studies the incidence of PEP has been evaluated in relation to the stadium (e.g. according to the Cambridge classification) of CP [23]. However, the patients in our cohort had rather early stage of chronic pancreatitis.~~

There are contradicting data on a non-dilated common bile duct as a risk factor for PEP [17, 21]. Our data are conclusive with a meta-analysis by Freeman et al. which did not show an increased risk by multivariate analysis. Sphincter oddi dysfunction, another previously described risk factor, was not examined in our patients [4].

Because most of the risk factors such as sex, age, bilirubin level, or bile duct diameter are not influenceable, efforts must be undertaken to minimize the procedure related risk factors and to optimize prophylactic measurements.

Inadvertent cannulation of the pancreatic duct was associated with an increased risk of PEP (odds ration ~~2.2~~ 7.468) in our study and others [17]. Procedures associated with cannulation of the pancreatic duct such as pre-cut papillotomy and injection of contrast agent into the pancreatic duct have also been confirmed as risk factors in other studies [5-7, 9, 10]. However, the reasons for inadvertent cannulation of the pancreatic duct are unclear. Our data show that difficult intubation of the papilla, which means more than 5 attempts, is a statistically significant risk factor for inadvertent cannulation. ~~This seems to happen more frequently during ERCPs on call. Although the reasons for that can be manifold, it is conceivable that ERCPs on call are likely more challenging or performed under difficult conditions.~~

**Conclusion**

Inadvertent cannulation of the pancreatic duct is a procedure related risk factor for PEP. Measurements on preventing inadvertent cannulation of the pancreatic duct should be established and studies on prophylactic measurements should focus particularly on patients with inadvertent cannulation of the pancreatic duct.

**References**

1. Andriulli A, Loperfido S, Napolitano G, Niro G, Valvano MR, Spirito F, et al. Incidence rates of post-ERCP complications: a systematic survey of prospective studies. Am J Gastroenterol. 2007;102(8):1781-8. doi: 10.1111/j.1572-0241.2007.01279.x. PubMed PMID: 17509029.

2. Kapral C, Muhlberger A, Wewalka F, Duller C, Knoflach P, Schreiber F, et al. Quality assessment of endoscopic retrograde cholangiopancreatography: results of a running nationwide Austrian benchmarking project after 5 years of implementation. Eur J Gastroenterol Hepatol. 2012;24(12):1447-54. doi: 10.1097/MEG.0b013e3283583c6f. PubMed PMID: 23114747.

3. Kochar B, Akshintala VS, Afghani E, Elmunzer BJ, Kim KJ, Lennon AM, et al. Incidence, severity, and mortality of post-ERCP pancreatitis: a systematic review by using randomized, controlled trials. Gastrointest Endosc. 2015;81(1):143-9 e9. doi: 10.1016/j.gie.2014.06.045. PubMed PMID: 25088919.

4. Chen YK, Foliente RL, Santoro MJ, Walter MH, Collen MJ. Endoscopic sphincterotomy-induced pancreatitis: increased risk associated with nondilated bile ducts and sphincter of Oddi dysfunction. Am J Gastroenterol. 1994;89(3):327-33. PubMed PMID: 8122639.

5. Loperfido S, Angelini G, Benedetti G, Chilovi F, Costan F, De Berardinis F, et al. Major early complications from diagnostic and therapeutic ERCP: a prospective multicenter study. Gastrointest Endosc. 1998;48(1):1-10. PubMed PMID: 9684657.

6. Dickinson RJ, Davies S. Post-ERCP pancreatitis and hyperamylasaemia: the role of operative and patient factors. Eur J Gastroenterol Hepatol. 1998;10(5):423-8. PubMed PMID: 9619390.

7. Masci E, Mariani A, Curioni S, Testoni PA. Risk factors for pancreatitis following endoscopic retrograde cholangiopancreatography: a meta-analysis. Endoscopy. 2003;35(10):830-4. doi: 10.1055/s-2003-42614. PubMed PMID: 14551860.

8. Sherman S, Ruffolo TA, Hawes RH, Lehman GA. Complications of endoscopic sphincterotomy. A prospective series with emphasis on the increased risk associated with sphincter of Oddi dysfunction and nondilated bile ducts. Gastroenterology. 1991;101(4):1068-75. PubMed PMID: 1889699.

9. Freeman ML, Nelson DB, Sherman S, Haber GB, Herman ME, Dorsher PJ, et al. Complications of endoscopic biliary sphincterotomy. N Engl J Med. 1996;335(13):909-18. doi: 10.1056/NEJM199609263351301. PubMed PMID: 8782497.

10. Johnson GK, Geenen JE, Johanson JF, Sherman S, Hogan WJ, Cass O. Evaluation of post-ERCP pancreatitis: potential causes noted during controlled study of differing contrast media. Midwest Pancreaticobiliary Study Group. Gastrointest Endosc. 1997;46(3):217-22. PubMed PMID: 9378207.

11. Sherman S, Lehman GA. ERCP- and endoscopic sphincterotomy-induced pancreatitis. Pancreas. 1991;6(3):350-67. PubMed PMID: 1713676.

12. Lesina M, Wormann SM, Neuhofer P, Song L, Algul H. Interleukin-6 in inflammatory and malignant diseases of the pancreas. Semin Immunol. 2014;26(1):80-7. doi: 10.1016/j.smim.2014.01.002. PubMed PMID: 24572992.

13. Bub H, Burner W, Riemann JF, Stolte M. Morphology of the pancreatic ductal epithelium after traumatization of the papilla of Vater or endoscopic retrograde pancreatography with various contrast media in cats. Scand J Gastroenterol. 1983;18(5):581-92. PubMed PMID: 6675180.

14. Testoni PA. Why the incidence of post-ERCP pancreatitis varies considerably? Factors affecting the diagnosis and the incidence of this complication. JOP. 2002;3(6):195-201. PubMed PMID: 12432186.

15. D. B. The etiology of post-ERCP pancreatitis. Gastrointestinal Endoscopy. 1989;35:189A.

16. Bradley EL, 3rd. A clinically based classification system for acute pancreatitis. Summary of the International Symposium on Acute Pancreatitis, Atlanta, Ga, September 11 through 13, 1992. Arch Surg. 1993;128(5):586-90. Epub 1993/05/01. PubMed PMID: 8489394.

17. Dumonceau JM, Andriulli A, Elmunzer BJ, Mariani A, Meister T, Deviere J, et al. Prophylaxis of post-ERCP pancreatitis: European Society of Gastrointestinal Endoscopy (ESGE) Guideline - updated June 2014. Endoscopy. 2014;46(9):799-815. Epub 2014/08/26. doi: 10.1055/s-0034-1377875. PubMed PMID: 25148137.

18. Committee ASoP, Anderson MA, Fisher L, Jain R, Evans JA, Appalaneni V, et al. Complications of ERCP. Gastrointest Endosc. 2012;75(3):467-73. doi: 10.1016/j.gie.2011.07.010. PubMed PMID: 22341094.

19. Freeman ML, Kozarek RA. Take 2 Indomethacin (Suppositories) and Call Me in the Morning? The Role of Nonsteroidal Anti-inflammatory Drugs in Protection Against Post-Endoscopic Retrograde Cholangiopancreatography Pancreatitis. Gastroenterology. 2016;150(4):805-8. Epub 2016/03/01. doi: 10.1053/j.gastro.2016.02.059. PubMed PMID: 26924086.

20. Thiruvengadam NR, Forde KA, Ma GK, Ahmad N, Chandrasekhara V, Ginsberg GG, et al. Rectal Indomethacin Reduces Pancreatitis in High- and Low-Risk Patients Undergoing Endoscopic Retrograde Cholangiopancreatography. Gastroenterology. 2016;151(2):288-97 e4. doi: 10.1053/j.gastro.2016.04.048. PubMed PMID: 27215656.

21. Freeman ML. Pancreatic stents for prevention of post-endoscopic retrograde cholangiopancreatography pancreatitis. Clin Gastroenterol Hepatol. 2007;5(11):1354-65. doi: 10.1016/j.cgh.2007.09.007. PubMed PMID: 17981248.

22. Freeman ML, DiSario JA, Nelson DB, Fennerty MB, Lee JG, Bjorkman DJ, et al. Risk factors for post-ERCP pancreatitis: a prospective, multicenter study. Gastrointest Endosc. 2001;54(4):425-34. PubMed PMID: 11577302.

23. Zhao ZH, Hu LH, Ren HB, Zhao AJ, Qian YY, Sun XT, et al. Incidence and Risk Factors for post-ERCP Pancreatitis in Chronic Pancreatitis. Gastrointest Endosc. 2017. doi: 10.1016/j.gie.2016.12.020. PubMed PMID: 28062312.

**Author Contributions**

VP and HA drafted and designed the study. MS and DH participated in study design and coordination. All authors were responsible for acquisition, analysis and interpretation of data. VP and HA drafted the manuscript. VP performed the statistical analysis. All authors reviewed and approved the final manuscript.
